# Hypermethylation Leads to Bone Morphogenetic Protein 6 Downregulation in Hepatocellular Carcinoma

**DOI:** 10.1371/journal.pone.0087994

**Published:** 2014-01-30

**Authors:** Yinghua He, Ying Cui, Baiying Xu, Jun Gu, Wei Wang, Xiaoying Luo

**Affiliations:** 1 State Key Laboratory of Oncogenes & Related Genes, Shanghai Cancer Institute, Renji Hospital, Shanghai Jiaotong University School of Medicine, Shanghai, China; 2 Guangxi Cancer Institute, Nanning, Guangxi, China; 3 Shanghai No. 6 People's Hospital, Medical School of Shanghai Jiaotong University, Shanghai, China; Institut national de la santé et de la recherche médicale, France

## Abstract

**Background:**

In the liver, bone morphogenetic protein 6 (BMP-6) maintains balanced iron metabolism. However, the mechanism that underlies greater BMP-6 expression in hepatocellular carcinoma (HCC) tissue than adjacent non-cancerous tissue is unclear. This study sought to investigate the epigenetic mechanisms of BMP-6 expression by analysing the relationship between the DNA methylation status of BMP-6 and the expression of BMP-6.

**Methods:**

Methylation-specific polymerase chain reaction (PCR), bisulphite sequencing PCR, the MethyLight assay, and quantitative real-time PCR were performed to examine BMP-6 methylation and mRNA expression levels. Immunohistochemistry (IHC) was performed on tissue arrays to evaluate the BMP-6 protein level.

**Results:**

BMP-6 mRNA expression was approximately 84.09% lower in HCC tissues than in adjacent non-cancerous tissues, and this low level of expression was associated with a poor prognosis. Moreover, the hypermethylation observed in HCC cell lines and HCC tissues was correlated with the BMP-6 mRNA expression level, and this correlation was validated following treatment with 5-aza-CdR, a demethylation agent. In addition, BMP-6 DNA methylation was upregulated by 68.42% in 114 clinical HCC tissue samples compared to adjacent normal tissues, whereas the BMP-6 staining intensity was downregulated by 77.03% in 75 clinical HCC tissue samples in comparison to adjacent normal tissues. Furthermore, elevated expression of BMP-6 in HCC cell lines inhibited cell colony formation.

**Conclusions:**

Our results suggest that BMP-6 CpG island hypermethylation leads to decreased BMP-6 expression in HCC tissues.

## Introduction

Hepatocellular carcinoma (HCC) is a highly aggressive tumour that can be rapidly fatal. Currently, HCC ranks as the sixth most common cause of cancer mortality worldwide [1]. This type of cancer is usually diagnosed at a stage when the disease is already advanced and incurable. In addition, the rate of tumour recurrence after curative liver resection is high, although surgery is the most effective treatment for HCC.

DNA methylation is one type of epigenetic modification, and aberrant methylation, consisting of DNA hypomethylation and/or promoter gene CpG hypermethylation, is implicated in the development of various solid tumours, including HCC. Cirrhosis [2], chronic hepatitis [3], and alcohol consumption [4] can induce liver lesions, and aberrant DNA methylation is associated with liver lesions, which indicates that such modifications could potentially mediate HCC. Previous research focusing on DNA methylation as a biomarker for the DNA methylome (microarray-based studies) has shown that there are differences in the levels of DNA methylation in various types of liver lesion (hepatitis, cirrhosis, and HCC, as well as normal liver tissue). Therefore, evaluating the status of DNA methylation could aid in determining the diagnosis, prognosis, and risk of carcinogenesis in HCC.

In humans, bone morphogenetic protein 6 (BMP-6) is encoded by the *BMP6* gene [5], [6]. BMPs are known for their ability to induce the growth of bone and cartilage, and BMP-6 can induce the expression of osteogenic markers in mesenchymal stem cells. Together, BMPs are a family of secreted signalling molecules that can induce ectopic bone growth, as well as part of the transforming growth factor-beta (TGF-β) superfamily. BMPs were originally identified through the ability of demineralised bone extract to induce endochondral osteogenesis in vivo at extraskeletal sites. Furthermore, based on its expression early in embryogenesis, BMP-6 has been proposed to have a role in early development.

In this study, we sought to identify genes that are regulated by DNA methylation based on the DNA methylome and mRNA profile in normal liver tissue and six HCC cell lines described previously [7]. We identified BMP-6 as a target gene and then validated the correlation between DNA methylation and BMP-6 expression in clinical tissues from HCC patients.

## Materials and Methods

### Patients and tissue microarray

Participants providing samples provided written informed consent to participate in this study, and the Ethics Committee of Shanghai Cancer Institute approved this study as well as the consent procedure. The tissue microarray study was also approved by the Ethics Committee. All research was performed on the mainland of China.

Paired tumour liver tissues and adjacent non-tumour liver tissues were collected from patients who underwent curative surgery for HCC at Guangxi Medical University, Guangxi Province, China. A diagnosis of HCC was confirmed by histological examination. The relevant clinical and pathological information was retrieved from the hospital database.

Glass slide tissue arrays for HCC were purchased from Shanghai Outdo Biotech Co. (Shanghai, China), and immunostaining (goat anti-BMP6, 1∶20, Santa Cruz Biotechnology, Santa Cruz, CA) was then performed on these tissue microarray slides. The staining was analysed according to the percentage of positively stained cells as well as the staining intensity by histopathology or using Image-Pro Plus 6.0 software (Media Cybernetics, Inc., Bethesda, MD).

### Cell culture and 5-aza-CdR treatments

The human HCC cell lines BEL-7402, Focus, PLC/PRF/5, QGY-7703, SMMC-7421, YY-8103, Hep3B, and Hep-1 (ATCC), as well as SMMC-7721 cells (Chinese Academy of Science, Shanghai, China), were maintained in DMEM supplemented with 10% foetal bovine serum in a humidified atmosphere of 95% air and 5% CO_2_ at 37°C. The SMMC-7721 and Hep3B cell lines were seeded at a density of 10^6^ cells/10-cm dish, cultured for 48 hours, and treated with freshly prepared 5 µM 5-aza-CdR (Sigma-Aldrich) dissolved in 50% acetic acid.

### DNA preparation and RNA extraction

Genomic DNA was isolated from cell lines using the QIAamp DNA Mini Kit (Qiagen, Valencia, CA) according to the manufacturer's protocol. Total RNA was isolated using the TRIzol Reagent for Molecular Biology (Invitrogen/Life Technologies). Genomic DNA from tumour samples was purified by standard phenol/chloroform purification. The DNA quality was verified by electrophoresis through an agarose gel and visualised with ethidium bromide.

### Bisulphite treatment and methylation-specific and bisulphite sequencing PCR analyses

Bisulphite conversion and polymerase chain reaction (PCR) analyses were performed as described previously [7]. The primers involved in methylation-specific (MSP) and bisulphite-sequencing PCRs (BSP) were designed using the following website: http://www.urogene.org/methprimer/index1.html. To design the primers, the target sequence from the region containing a methylation signal in the DNA methylome was used, considering the repeat sequence around the target sequence. The following primers were used for the BSP assay: BMP6bspf, 5′- GAAGGGTGTAGAGAAGATTTTTTTT-3′; and BMP6bspt, 5′-TACTCACCCACTTCAAAACCTAAAC-3′. The following primers were used for the MSP assay: Msp forward methylation primer, 5′-GGAGGGACGCGTTGTTAGC-3′; and Msp reverse methylation primer, 5-CGTAAAACCCCACCCCG-3′. Regarding the reaction conditions, the melting temperature (Tm) was 52°C, and 35 PCR cycles were performed.

### M.SssI methylation assay

DNA from the SMMC-7721 HCC cell line was used as a substrate for M.SssI treatment. The DNA (0.05 µg/µl) was incubated with M.SssI at a concentration of 1 U/µg DNA (0.05 U/µl) and 0.16 mM AdoMet overnight at 37°C. Next, additional AdoMet (to 0.20 mM) and M.SssI (to 0.065 U/µl) were added, followed by a second overnight incubation at 37°C. The sample was stored at 4°C, and 18-µl aliquots (containing 0.9 µg DNA) were used for bisulphite conversion and recovery, as described above [8].

### The MethyLight assay

PCR was performed using a 96-well optical tray with caps at a final reaction volume of 20 µl. Samples contained 8 µl of Real MasterMix (Taqman; Tiangen), 1 µl of bisulphite-treated DNA, 250 nM of each of the primers, and 125 nM of FAM-labelled probes. The modified DNA was amplified via the MethyLight real-time PCR reaction using the TaqMan gene assay and the 7500/7500 Fast Real-Time PCR System (Applied Biosystems, Foster City, CA, USA). The forward primer and reverse primer of the BMP6 MSP assay were used in this assay, 5′- CGCGCAACTACAACCATCCCG -3′, was used in this assay as the probe for BMP6 and the primers/probe for the internal control (Alu-C4) have been described previously [8]. Each PCR program consisted of initial denaturation (95°C, 10 min), 45 cycles of denaturation (95°C, 15 s), and annealing/extension (60°C, 1 min). The methylation ratio was determined according to the absolute quantification results of the real-time PCR. The quantity of the amplified target genes in the test samples was normalised to that of Alu-C4 to measure the levels of input DNA. Additionally, DNA treated with M.SssI served as a methylated reference. The amount of methylated DNA (PMR, percentage of methylated reference) at the BMP6 locus was calculated by dividing the GENE:Alu-C4 ratio of a sample by the GENE: Alu-C4 ratio of SssI-treated human genomic DNA (presumably fully methylated) and then multiplying by 100.

### Real-time quantitative RT-PCR for mRNA expression

Total RNA was extracted using the TRIzol reagent (Invitrogen, Carlsbad, CA) according to the manufacturer's protocol. cDNA was then reverse-transcribed from 1 µg of RNA using a SYBR®Prime Script™ RT-PCR kit (Takara Biochemicals, Tokyo, Japan). The reactions were performed in an ABI PRISM®7900HT Real-Time PCR System. The thermal cycling conditions were as follows: an initial cycle of 95°C for 15 s, followed by 40 cycles of 95°C for 5 s and 60°C for 30 s. Each experiment was performed in a 20-µl reaction volume containing 10 µl of SYBR® Prime Ex Taq™ II (2×), 0.8 µl of forward primer and reverse primer (10 µM each), 0.4 µl of ROX Reference Dye or Dye II (50×), 2 µl of cDNA, and 6 µl of H_2_O. β-Actin was chosen as an internal control. The quantification of mRNA was calculated using the comparative Ct (the threshold cycle) method with the following formula: Ratio = 2^−ΔΔct^ = 2^−[ΔCt(sample)−ΔCt(calibrator)]^, where ΔCt is equal to the Ct of the target genes minus the Ct of the endogenous control gene (β-actin). The primers for BMP6 (BMP6RTF: 5′-AGCGTGGTGACAAGGGATG-3′; BMP6RTR: 5′-TGGACCTCACTCACTTTGAAGA-3′) were designed using the following website: http://pga.mgh.harvard.edu/primerbank/:. The internal control actin primers were designed as described previously.

### Cell proliferation and colony formation assays

Cell proliferation was determined using WST-8 staining with the Cell Counting Kit-8 (Dojinodo, Shanghai, China) according to the manufacturer's instructions. For colony formation assays, 500 cells were plated onto 6-well plates and incubated at 37°C for 2 weeks. Cells were then stained with crystal violet, and the numbers of colonies per well were counted.

### Western blot analysis

Immunoblotting experiments were performed according to standard procedures. The following antibodies were used: BMP-6 antibody (1∶50; Santa Cruz Biotechnology, Santa Cruz, CA), FLAG antibody (1∶2,000; Sigma-Aldrich St. Louis, MO), and GAPDH antibody [used as the internal control (1∶10,000; Kang-Chen Bio-tech Shanghai, China)].

### Statistical analysis

All experiments were repeated 3 times. Data are presented as the mean ± standard deviation (SEM) and were analysed using Student's *t*-test. P values less than 0.05 were considered statistically significant. Statistical analyses were performed using GraphPad Prism 3.02 (GraphPad Software Inc., San Diego, CA).

### Accession numbers

BMP6: NM_001718

## Results

### BMP-6 mRNA expression is downregulated in HCC tissue and is correlated with patient outcomes

**Figure 1:**
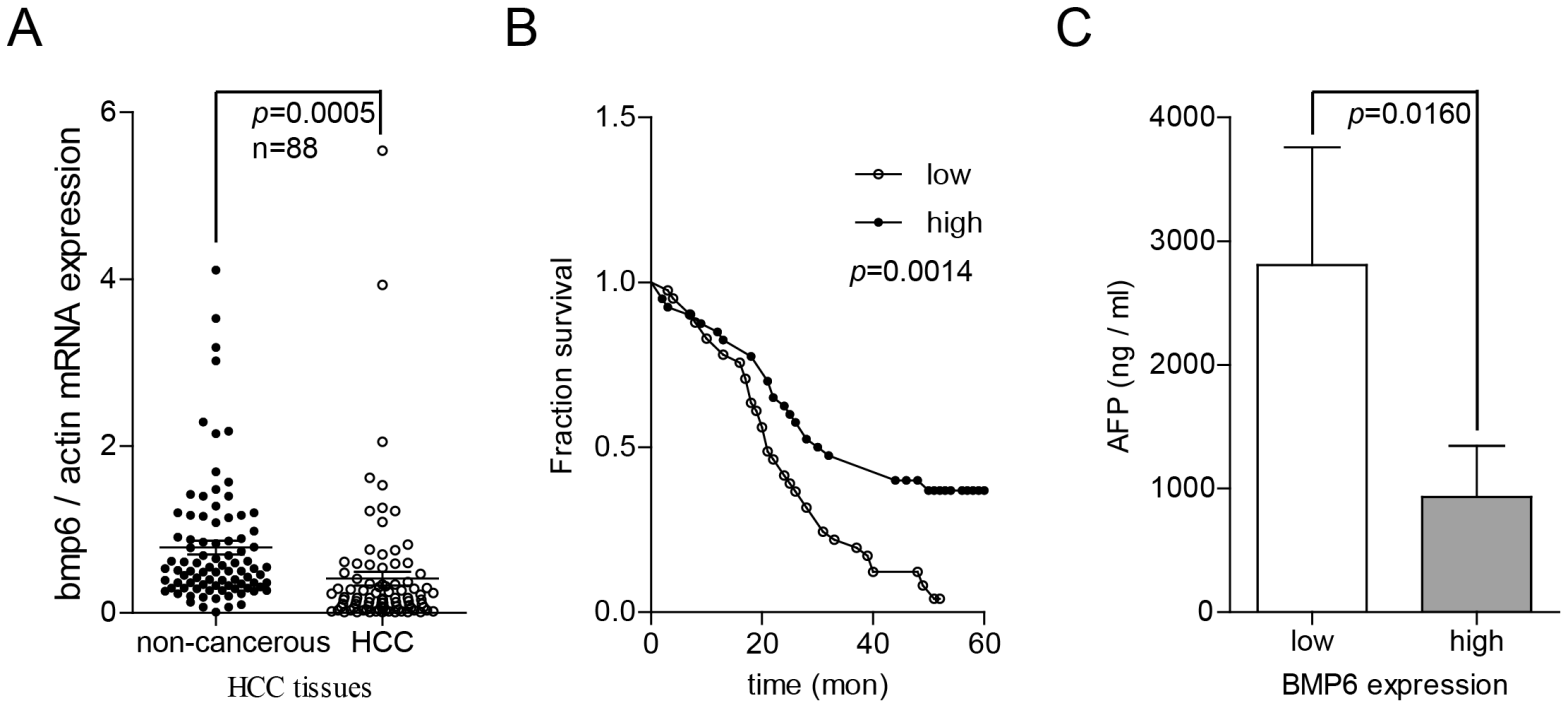
The low expression level of BMP-6 in HCC tissues is associated with a poor prognosis. (A) We analysed the expression levels of BMP-6 mRNA in 88 pairs of HCC tissues and found an 85.85% decrease in the expression level of BMP-6 in HCC tissues compared to adjacent non-cancerous tissues [*p* = 0.0005 (paired *t* test)]. BMP-6 expression was downregulated in 84.09% of these 88 pairs of tissues. (B) Downregulation of BMP-6 was associated with poor prognosis. BMP-6 expression in HCC tissues was grouped from lowest to highest; the 44 HCC tissues with the lowest BMP-6 expression levels were placed into the low-expression group, whereas the 44 HCC tissues with the highest BMP-6 expression levels were placed into the high-expression group. (C) Downregulation of BMP-6 was associated with a high AFP level.

We analysed 88 pairs of HCC clinical samples and found that the BMP-6 levels in HCC tissues were downregulated by 85.85% (0.1252/0.8846; with the BMP-6 level in normal liver tissue designated as 1 in comparison to the adjacent non-cancerous tissue. The rate of decreased BMP-6 expression was 84.09% (74/88) (Figure 1A). Next, we categorised the HCC samples into 2 groups based on their expression of BMP-6; the 44 samples with lower expression levels were placed into the low-expression group, and the 44 samples with higher expression levels were placed into the high-expression group. We evaluated patient survival times following treatment, and these are shown by the survival curve in Figure 1B. The results indicated that the HCC patients with low BMP-6 expression had poorer prognoses than those with high BMP-6 expression (*p* = 0.0014). The level of AFP also showed a significant difference between these groups, as the AFP level was higher in the high BMP-6 expression group of HCC patients than in the low BMP-6 expression group (*p* = 0.0160).

These results demonstrated that HCC tissues had low BMP-6 expression and that patients with low BMP-6 expression had poor prognoses. Additionally, BMP-6 showed a negative correlation with the AFP level, suggesting that BMP-6 may be a potential diagnostic marker.

### The BMP6 promoter is hypermethylated and HCC cell lines show decreased BMP-6 expression

**Figure 2:**
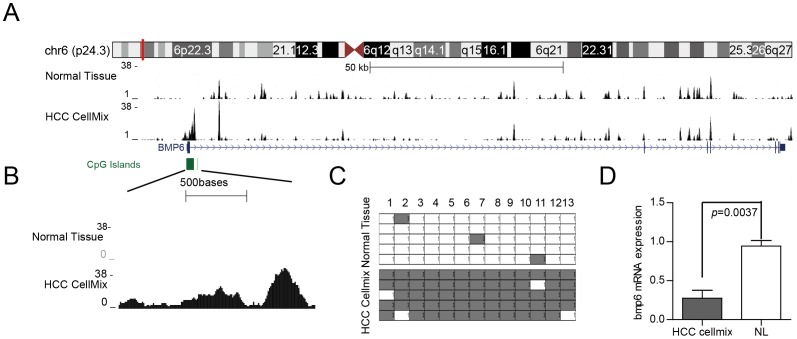
The HCC cell mix showed hypermethylation of the BMP-6 promoter and low mRNA expression of BMP-6. (A) BMP-6 is located on chromosome 6, and there is a CpG island in the promoter of BMP-6 with a DNA methylation signal. The DNA methylation signal of the BMP-6 gene body was decreased in the HCC cell mix compared to normal liver tissue. (B) Enlarged region of the CpG island within the BMP-6 promoter. There was a significant DNA methylation signal in the HCC cell mix but not in normal liver tissue. (C) Hypermethylation of the BMP-6 promoter in HCC cells was detected according to the BSP-based assay. The levels of DNA methylation were 4.62% and 93.85% in normal liver tissue and the HCC cell mix, respectively. BMP-6 expression levels in normal liver tissue and the HCC cell mix were analysed by qRT-PCR. The BMP-6 expression level was analysed in each sample 3 times, and then the BMP-6 expression level in the HCC cell mix was analysed in 6 cell lines 3 times followed by calculation of the average value from the 6 cell lines. All mRNA expression levels were normalised to actin mRNA expression levels. These results were analysed using Student's *t*-test.

A CpG island (or CpG-rich sequence) is located within the BMP-6 promoter, and previously obtained DNA methylome data ([7]) showed that a DNA methylation signal is also located in this region (Figure 2A). This DNA methylation signal is decreased in the BMP-6 gene body, which is consistent with the typical DNA methylation signal observed in cancer tissue. When we amplified the CpG locus within the BMP-6 promoter, the data showed a strong DNA methylation signal at this locus in HCC cells but no DNA methylation signal in normal liver tissue (Figure 2B). Next, we evaluated the DNA methylation status of this region using a BSP-based assay, and the results indicated that there were DNA methylation differences between HCC and normal liver tissue (Figure 2C). In addition, qRT-PCR analysis was performed to assess BMP-6 expression in normal liver tissue and HCC cells (Figure 2D), and lower BMP-6 expression was observed in the BMP-6 hypermethylated HCC cell mix.

Together, the results of these assays showed that there was hypermethylation at the BMP-6 CpG island within the promoter and that this methylation signal was associated with HCC tissue in comparison to normal liver tissue.

### DNA methylation status of the CpG island in the BMP-6 promoter and expression of BMP-6 expression in HCC cells following treatment with 5-aza-CdR

**Figure 3:**
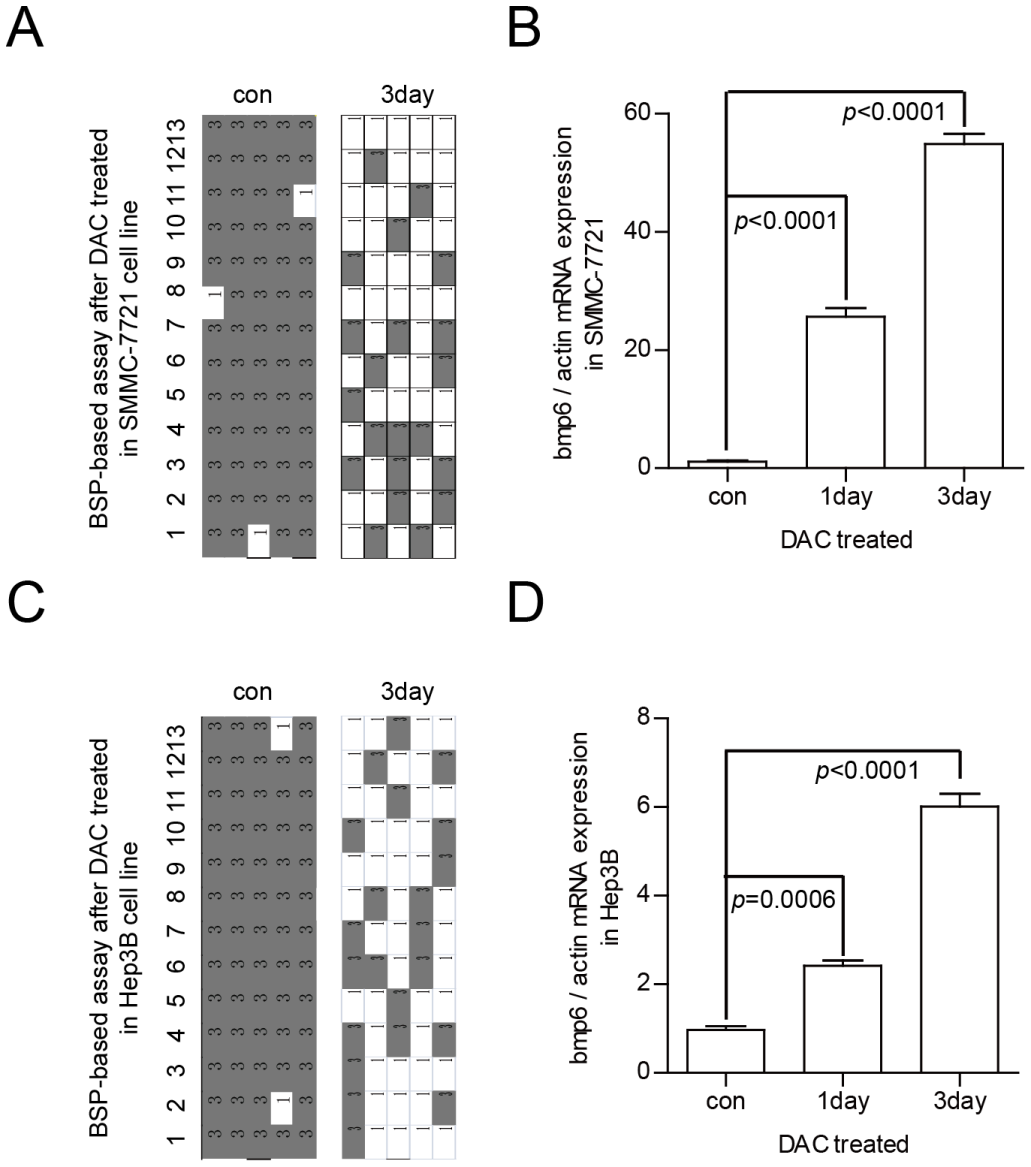
Alterations in the DNA methylation status and BMP-6 mRNA expression in HCC cell lines treated with 5′-aza-CdR. The BMP-6 locus became hypomethylated following 5′-aza-CdR treatment. In SMMC-7721 cells, the rate of DNA methylation prior to treatment was 95.38%, and this decreased to 32.31% after 3 days of treatment (A); In Hep3B cells, the rate of DNA methylation prior to treatment was 96.92%, and this decreased to 33.85% after 3 days of treatment (C). The levels of BMP-6 expression were upregulated following treatment with 5′-aza-CdR in SMMC-7721 cells (B) and Hep3B cells (D). The status of DNA methylation was evaluated with the BSP-based assay, and the expression level of BMP-6 was assessed by qRT-PCR. These results were analysed using the Student's *t*-test.

To confirm the correlation between the BMP-6 DNA methylation status and BMP-6 expression level, we used SMMC-7721 and Hep3B cells as models. In the SMMC-7721 cell line, the rate of DNA methylation decreased from 95.38% to 32.31% after 3-day treatment with 5-aza-CdR (Figure 3A). Furthermore, the qRT-PCR assays revealed that BMP-6 expression in 5-aza-CdR-treated SMMC-7721 cells was upregulated (Figure 3B) in comparison to the untreated controls. Additionally, in the Hep3B cell line, the rate of DNA methylation decreased from 96.92% to 33.85% after 3-day treatment with 5-aza-CdR (Figure 3C), and the qRT-PCR assays revealed that BMP-6 expression in 5-aza-CdR-treated Hep3B cells was upregulated (Figure 3D) in comparison to the untreated controls. Identical results were observed in independent triplicate assays.

These results show that following 5-aza-CdR-mediated demethylation of the CpG island within the BMP-6 promoter, BMP-6 expression is upregulated. Therefore, DNA methylation can regulate BMP-6 expression.

### DNA methylation status of the CpG island in the BMP-6 promoter and BMP-6 expression in HCC cells in HCC primary tumours

**Figure 4:**
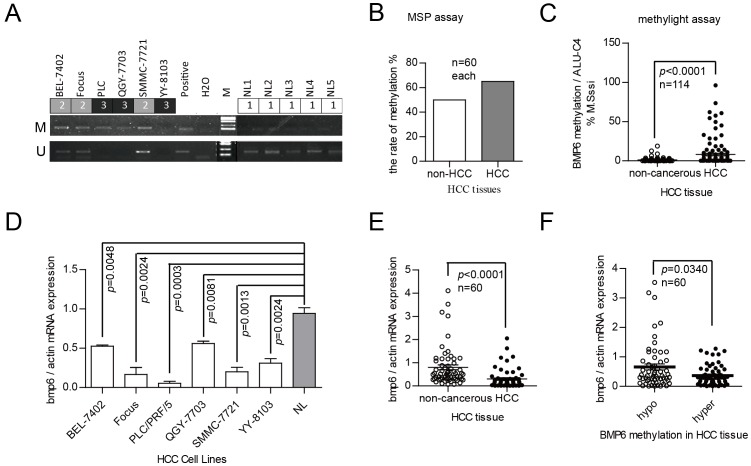
The BMP-6 promoter is hypermethylated, and BMP-6 is expressed at a low level in HCC tissues. (A) An MSP-based assay was used to detect BMP-6 promoter methylation in 6 HCC cell lines and normal liver tissues. No methylation of the BMP-6 promoter was observed in 5 normal liver tissues. (B) An MSP-based assay was used to detect BMP-6 promoter methylation in 60 pairs of clinical HCC tissues. BMP-6 promoter methylation was demonstrated in 65% of HCC tissues, whereas this rate in the adjacent non-cancerous tissue was 50%. (C) The Taqman probe-based MethyLight assay was used to detect BMP-6 promoter methylation in 114 pairs of clinical HCC tissues, and the level of DNA methylation was upregulated by 23.44 fold in HCC tissues compared to adjacent non-cancerous tissues. (D) qRT-PCR was used to detect BMP-6 mRNA expression in normal liver tissue and HCC cells. The HCC cell lines used are shown, and the normal liver tissue was the NL1 sample shown in Figure 4A. All BMP-6 expression levels in HCC cell lines were lower than those in normal liver tissue. (E) qRT-PCR was used to detect the BMP-6 mRNA expression in 60 pairs of HCC clinical samples, containing both DNA and RNA samples. We obtained similar results as those in Figure 1A. (F) The expression level of BMP-6 was correlated with BMP-6 promoter methylation. The level of BMP-6 DNA methylation obtained according to the MethyLight assay in 60 pairs HCC tissues is listed from lowest to highest. The 60 tissues with the lowest DNA methylation levels were placed into the hypomethylation group, whereas the 60 tissues with the highest DNA methylation levels were placed into the hypermethylation group. The HCC patients with BMP-6 hypermethylation had low levels of BMP-6 mRNA. The results were analysed using the Student's *t*-test.

Next, we performed MSP assays to detect the DNA methylation status of the CpG island within the BMP-6 promoter in normal liver tissues and HCC cell lines. The results of this analysis are shown in Figure 4A; all DNA was hypermethylated in HCC cells but hypomethylated in normal tissues. Furthermore, BMP-6 expression was correlated with the methylation status in these samples (Figure 4D).

We then evaluated the status of DNA methylation in 60 pairs of HCC tissues and the adjacent non-cancerous tissues using the MSP assay. The results of this analysis are shown in Figure 4B; the data indicated that 65% of these HCC tissues showed hypermethylation (39/60), whereas 50% of the adjacent non-cancerous tissues showed hypermethylation (30/60).

Furthermore, we assessed 114 pairs of HCC tissues and adjacent non-cancerous tissues using the quantitative MethyLight assay. The results of this analysis are shown in Figure 4C. These data show that the extent of BMP-6 DNA methylation in HCC tissues was upregulated by 23.44 fold (2.6628/0.1136; BMP-6 DNA methylation level in the M. SssI sample designated as 100) in comparison to adjacent non-cancerous tissue. The rate of increased BMP-6 DNA methylation was 68.42% (78/114).

In 60 of 114 sample pairs, BMP-6 mRNA expression (total clinical sample number was 88, 60 of 88 smaple pairs have DNA methylation data) was downregulated in HCC tissue compared to adjacent non-cancerous tissue (Figure 4E). We then placed these samples into 2 groups based on their BMP-6 DNA methylation levels and compared the BMP-6 mRNA expression levels in these samples. The results of this analysis are shown in Figure 4F and revealed that HCC patients with BMP-6 hypermethylation had low levels of BMP-6 mRNA expression (BMP-6 mRNA expression was reduced to 19.06%; *p* = 0.0340).

These results indicated that hypermethylation at the CpG island of the BMP-6 promoter was correlated with BMP-6 mRNA expression in clinical HCC samples.

### BMP-6 protein expression is downregulated in HCC tissue and is correlated with patient outcome

**Figure 5:**
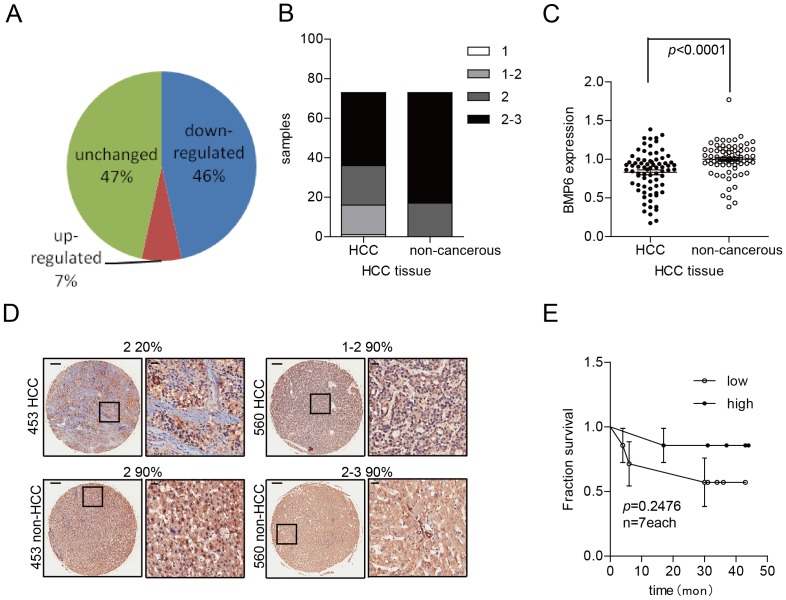
The BMP-6 IHC staining intensity is downregulated in HCC tissue. IHC was performed in 75 pairs of HCC tissue arrays. (A) The staining intensity of BMP-6 in HCC tissues was downregulated by 46% compared to adjacent non-cancerous tissues. Additionally, the rate of upregulation was 7%. The staining intensity was confirmed by histopathology. (B) The staining intensity score was lower in HCC tissue compared to adjacent non-cancerous tissue. Sixteen samples of HCC tissue scored 1 or 1–2, whereas these scores were not observed in the adjacent non-cancerous tissue samples; furthermore, scores of 2–3 emerged 37 times in HCC tissue samples as compared to 56 times in adjacent non-cancerous tissue samples. (C) The BMP-6 staining intensity was downregulated, as assessed using Image-Pro 6.0 software. The staining intensity was downregulated to 83.14% in HCC tissues compared to adjacent non-cancerous tissues. (D) The representative image shows that the BMP-6 staining intensity was downregulated in relation to the rate of cell staining or the direct staining intensity. (E) The BMP-6 staining intensity likely correlated with poor prognosis; however, in 75 pairs of HCC tissues, only 14 pairs of HCC clinical samples had outcome data available.

To better evaluate BMP-6 expression in HCC tissues, we examined the expression of BMP-6 in 75 pairs of HCC tissue arrays. Immunohistochemical (IHC) staining showed that BMP-6 expression was decreased in 46% of HCC cases (34 of 75), BMP-6 expression was unchanged in 47% of HCC cases (35/75), and BMP-6 expression was increased in 7% of HCC cases (6 of 75) (Figure 5A). Furthermore, the BMP-6 staining score showed that the values obtained for HCC tissues were lower in comparison to those of the adjacent normal tissue (Figure 5B).

We then quantified the intensity of BMP-6 expression by IHC using Image-Pro Plus 6.0 software. The results of this analysis are shown in Figure 5C, and these data showed that the values of BMP-6 staining intensity in HCC tissues were downregulated by 16.86% (100/120.27; the BMP-6 IHC intensity average level in HCC samples was designated as 100) in comparison to adjacent normal tissue. The rate of decreased BMP-6 IHC intensity was 77.03% (57/75) (Figure 5C). Additionally, the BMP-6 IHC staining intensity in HCC tissues corresponded to full BMP-6 downregulation (Figure 5D, No. 560) or the percentage of positively stained cells that exhibited downregulation (Figure 5D, No. 453). In these 75 pairs of HCC tissues, we evaluated the survival times of 14 patients after treatment, and we placed the samples into 2 groups based on the BMP-6 IHC staining intensity levels in HCC tissues. The survival curves are shown in Figure 5E (*p* = 0.2470) and demonstrate that the HCC patients with low BMP-6 IHC staining intensity had poorer prognoses.

These results show that BMP-6 protein expression was downregulated in HCC clinical samples.

### Overexpression of BMP-6 inhibits colony formation in HCC cell lines

**Figure 6:**
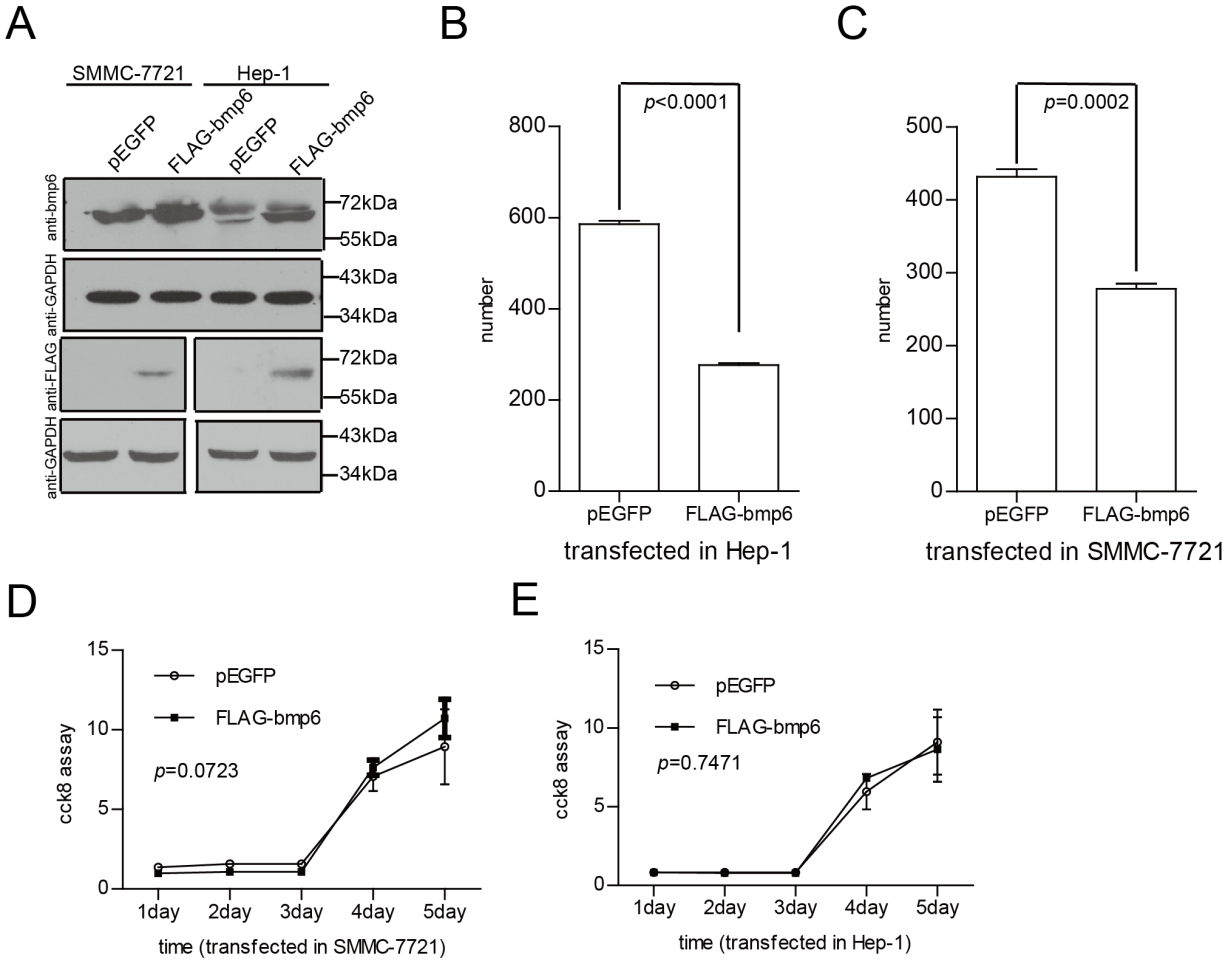
Overexpression of BMP-6 in HCC cell lines inhibits colony formation. (A) We cotransfected the BMP-6 overexpression construct into SMMC-7721 and Hep-1 cells, and BMP-6 overexpression was confirmed by Western blotting using anti-BMP-6 and anti-FLAG antibodies. Overexpressed BMP-6 inhibited colony formation in SMMC-7721 (B) and Hep-1 cells (C); however, BMP-6 overexpression did not influence cell growth in these 2 cell lines (D and E).

To better understand the biological functions of BMP-6 in the development of HCC, we first constructed a vector expressing BMP-6 and then transfected this vector into the SMMC-7721 and Hep-1 HCC cell lines. We then evaluated BMP-6 protein levels in these cell lines using BMP-6 and FLAG antibodies (Figure 6A). In cell proliferation and colony formation assays, the overexpression of BMP-6 had a clear impact on in vitro HCC cell colony formation in Hep-1 (Figure 6B) and SMMC-7721 cells (Figure 6C); however, overexpression of BMP-6 showed no clear impact on in vitro HCC cell proliferation using the CCK-8 assay in Hep-1 (Figure 6E) and SMMC-7721 cells (Figure 6D).

These results show that overexpression of BMP-6 inhibited colony formation in HCC cell lines.

## Discussion

DNA methylation is an important step in cancer development. In this study, we observed DNA methylation of the BMP-6 promoter based on next-generation sequencing of the MBD affinity DNA methylation sequence. The technology associated with next-generation sequencing has advanced human methylome analysis from single chromosomes to low-resolution (100 bp) complete genomes to single-base-resolution complete genomes. Using this technology, we obtained the DNA methylomes of normal liver tissue and HCC cell lines to carefully analyse the DNA methylation status of HCC. Furthermore, we integrated the analysis of methylome data with mRNA expression microarray data (these -omics investigations were described previously) using BMP-6 as a candidate gene.

Many types of solid tumours demonstrate elevated expression levels of BMP-6 [9]–[13], whereas other types of tumours display reduced BMP-6 expression levels [14], [15]. In addition, although many studies have shown that BMP-6 promotes cell migration, cell invasion, proliferation, and colony formation [13], [16], [17], many other studies have shown that BMP-6 inhibits cell migration, cell invasion, proliferation, and colony formation [14], [18]–[20]. Thus, the function of BMP-6 in tumours remains controversial, and BMP-6 likely exerts its function according to the specific conditions.

Upon comparing 16 samples of HCC tissue to 13 samples of normal liver tissue, the level of BMP-6 expression was upregulated, and BMP-6 was shown to inhibit iron metabolism by downregulating hemojuvelin expression [21]. However, differences in BMP-6 expression between HCC and adjacent non-cancerous tissue were not clear, and the factors regulating BMP-6 expression and its function were not addressed.

In 2009, a study showed that BMP-6 was a key regulator of hepcidin, a small peptide secreted by the liver that regulates iron metabolism in mammals [22]. Furthermore, in gene knockout mice, the lack of BMP-6 induced massive iron overload [22], [23]. Thus, BMP-6 is an important protein in the liver for maintaining balanced iron metabolism.

In this study, we compared BMP-6 mRNA expression in 88 pairs of HCC tissue and the corresponding adjacent normal tissue, as well as the BMP-6 staining intensity (the protein level) in 75 pairs of HCC tissue and adjacent normal tissue, to definitively show that BMP-6 expression is downregulated in HCC.

Previous studies have shown that the DNA methylation signal of BMP-6 promoter can be used as a biomarker [23]–[25]. In this study, we found that DNA methylation regulated BMP-6 mRNA expression using conventional MSP and MethyLight assays.

In conclusion, our findings indicate that hypermethylation drives the downregulation of BMP-6 in HCC. At the mRNA level, BMP-6 is downregulated, and this downregulation is associated with poor patient outcomes. At the DNA methylation level, this signal is upregulated in HCC samples, whereas at the protein level, the BMP-6 staining intensity is downregulated in HCC tissues. Based on the results of the BSP assay (DAC treatment induced demethylation and upregulation of BMP-6 mRNA, and there was a correlation between DNA methylation and BMP-6 mRNA expression), DNA methylation likely drives BMP-6 expression. Taken together, these results show that hypermethylation downregulates BMP-6 in HCC, BMP-6 maybe as a prognosis marker.
